# (*E*)-3-Methyl-2,6-di­phenyl­piperidin-4-one *O*-(3-methyl­benzo­yl)oxime

**DOI:** 10.1107/S1600536814016638

**Published:** 2014-07-31

**Authors:** V. Kathiravan, K. Gokula Krishnan, T. Mohandas, V. Thanikachalam, P. Sakthivel

**Affiliations:** a Department of Physics, Goverment Arts College, Karur 639 005, India; b Department of Chemistry, Annamalai University, Annamalainagar, Chidambaram, India; c Department of Physics, Shri Angalamman College of Engineering and Technology, Siruganoor, Tiruchirappalli, India; d Department of Physics, Urumu Dhanalakshmi College, Tiruchirappalli 620 019, India

**Keywords:** crystal structure, piperidinone, oxime, hydrogen bonding

## Abstract

In the title compound, C_26_H_26_N_2_O_2_, the piperidine ring exhibits a chair conformation. The phenyl rings are attached to the central heterocycle in an equatorial position. The dihedral angle between the planes of the phenyl rings is 57.58 (8)°. In the crystal, C—H⋯O inter­actions connect the mol­ecules into zigzag chains along [001].

## Related literature   

For the biological activity of oxime esters, see: Crichlow *et al.* (2007 ▶); Hwu *et al.* (2008 ▶); Neely *et al.* (2013 ▶); Liu *et al.* (2011 ▶). For ring conformations, see: Cremer & Pople (1975 ▶). For comparable structures, see: Park *et al.* (2012*a* ▶,*b* ▶).
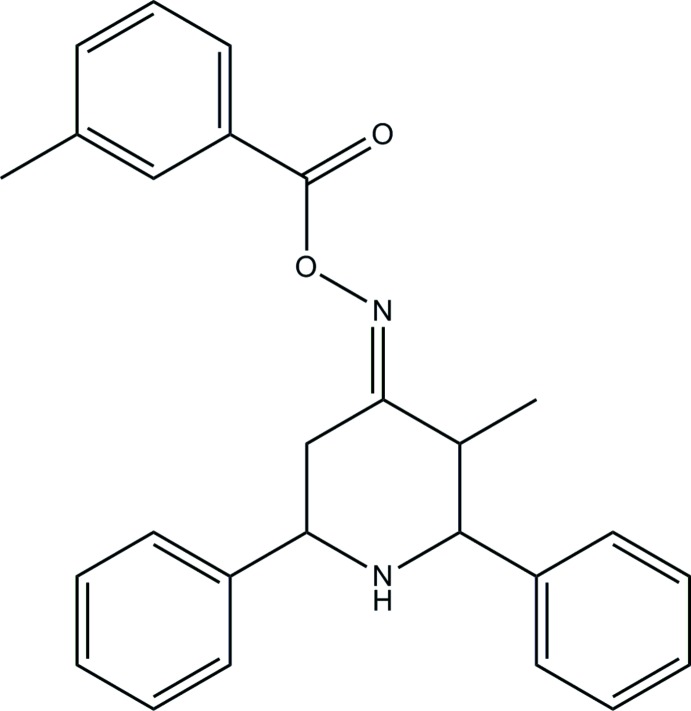


## Experimental   

### 

#### Crystal data  

C_26_H_26_N_2_O_2_
*M* *_r_* = 398.49
Monoclinic, 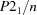

*a* = 10.6265 (6) Å
*b* = 12.7146 (7) Å
*c* = 16.4031 (8) Å
β = 99.524 (2)°
*V* = 2185.7 (2) Å^3^
*Z* = 4
Mo *K*α radiation
μ = 0.08 mm^−1^
*T* = 293 K
0.30 × 0.25 × 0.20 mm

#### Data collection  

Bruker Kappa APEXII CCD diffractometer
Absorption correction: multi-scan (*SADABS*; Bruker, 2008 ▶) *T*_min_ = 0.977, *T*_max_ = 0.985
37978 measured reflections
5367 independent reflections
3097 reflections with *I* > 2σ(*I*)
*R*_int_ = 0.034

#### Refinement  

*R*[*F* ^2^ > 2σ(*F* ^2^)] = 0.053
*wR*(*F* ^2^) = 0.175
*S* = 1.04
5367 reflections
275 parameters
H atoms treated by a mixture of independent and constrained refinement
Δρ_max_ = 0.25 e Å^−3^
Δρ_min_ = −0.23 e Å^−3^

### 

Data collection: *APEX2* (Bruker, 2008 ▶); cell refinement: *SAINT* (Bruker, 2008 ▶); data reduction: *SAINT* (Bruker, 2008 ▶); program(s) used to solve structure: *SHELXS97* (Sheldrick, 2008 ▶); program(s) used to refine structure: *SHELXL97* (Sheldrick, 2008 ▶); molecular graphics: *ORTEP-3 for Windows* (Farrugia, 2012 ▶) and *Mercury* (Macrae *et al.*, 2008 ▶); software used to prepare material for publication: *SHELXL97* (Sheldrick, 2008 ▶) and *PLATON* (Spek, 2009 ▶).

## Supplementary Material

Crystal structure: contains datablock(s) global, I. DOI: 10.1107/S1600536814016638/bt6984sup1.cif

Structure factors: contains datablock(s) I. DOI: 10.1107/S1600536814016638/bt6984Isup2.hkl

Supporting information file. DOI: 10.1107/S1600536814016638/bt6984Isup3.cml

CCDC reference: 1005453

Additional supporting information:  crystallographic information; 3D view; checkCIF report

## Figures and Tables

**Table 1:** Hydrogen-bond geometry (Å, °)

*D*—H⋯*A*	*D*—H	H⋯*A*	*D*⋯*A*	*D*—H⋯*A*
C3—H3⋯O2^i^	0.93	2.59	3.485 (3)	160

Symmetry code: (i) 
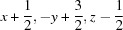
.

**Fig. 1.**
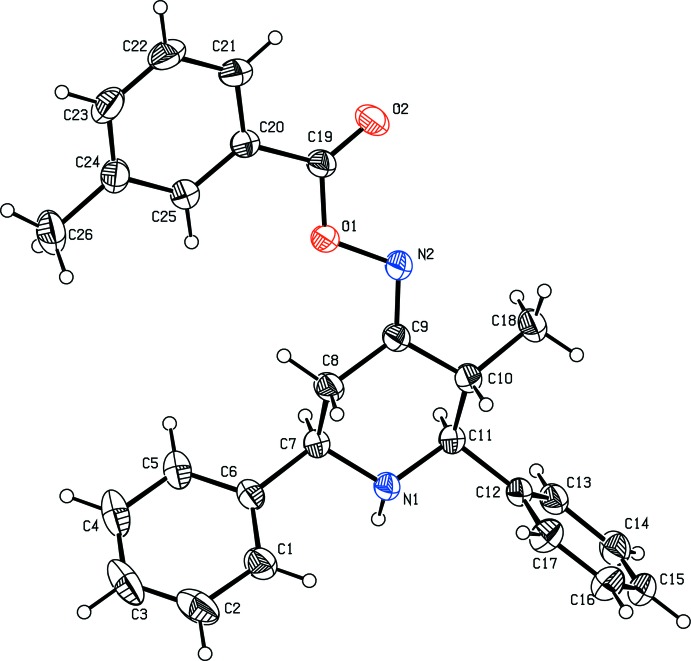
The molecular structure of the title compound with the atom numbering scheme, displacement ellipsoids are drawn at 30% probability level. H atoms are present as small spheres of arbitary radius.

**Fig. 2.**
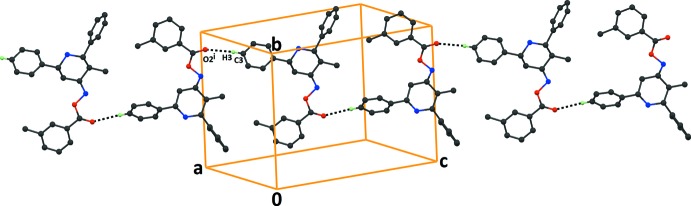
Part of crystal structure of the title compound, showing the formation one dimensional C(12) chains running parallel to [0 0 1] axis.

## supplementary crystallographic information

### S1. Comment

The chemistry of oxime esters are serving as important synthetic intermediate, and have been employed as starting materials for both synthetic and medicinal chemistry (Crichlow *et al.* 2007; Hwu *et al.*2008; Neely *et al.*2013). Oxime esters have received great potential in biologically active molecules such as agrochemical industries (Liu *et al.*, 2011).

The central ring (N1/C7/C8/C9/C10/C11) adopts a chair conformation with the puckering parameters Q=0.5398 Å, θ=8.88° and φ=30.1509° (Cremer & Pople, 1975).

The bond distances and bond angles in the title compound agree very well with the corresponding values reported in closely related compounds (Park *et al.*, 2012a,b).

This stucture was stabilized by C—H···O intramolecular interactions linking the molecules to zigzag chains running parallel to [001] axis.

### S2. Experimental

A mixture of 3-methyl-2,6-diphenylpiperidin-4-one oxime (0.73 g, 2.5 mmol) and *m*-methylbenzoic acid (0.37 g, 2.75 mmol) in dry pyridine (7 ml) was stirred at ambient temperature. POCl_3_ (0.25 ml, 2.75 mmol) was added drop wise to the reaction mixture and stirring is continued for 20 to 30 min. The progress of the reaction was monitored by TLC. After completion of the reaction, a saturated solution of NaHCO3 was added portion wise to the reaction mixture and the crude product was thrown out as a precipitate. The crude product was then recrystallized from absolute ethanol to get the pure 3-methyl-2,6-diphenylpiperidin-4-one-O-(3-methylbenzoyl) oxime. Yield 0.76 g (78%).

### S3. Refinement

The positions of the hydrogen atoms were identified from difference electron density maps. The hydrogen atoms bound to the C atoms are treated as riding atoms, with d(C—H)=0.93 and *U*iso(H) = 1.2*U*eq(C) for aromatic, d(C—H)=0.97 and *U*iso(H)=1.2*U*eq(C) for methylene and d(C—H)=0.96 and *U*iso(H) =1.5*U*eq(C) for methyl groups. The H atom bonded to N was freely refined.

### Crystal data

**Table:**

C_26_H_26_N_2_O_2_	*F*(000) = 848
*M**_r_* = 398.49	*D*_x_ = 1.211 Mg m^−^^3^
Monoclinic, *P*2_1_/*n*	Mo *K*α radiation, λ = 0.71073 Å
Hall symbol: -P 2yn	Cell parameters from 3910 reflections
*a* = 10.6265 (6) Å	θ = 2.0–28.3°
*b* = 12.7146 (7) Å	µ = 0.08 mm^−^^1^
*c* = 16.4031 (8) Å	*T* = 293 K
β = 99.524 (2)°	Block, colourless
*V* = 2185.7 (2) Å^3^	0.30 × 0.25 × 0.20 mm
*Z* = 4	

### Data collection

**Table:**

Bruker Kappa APEXII CCD diffractometer	5367 independent reflections
Radiation source: fine-focus sealed tube	3097 reflections with *I* > 2σ(*I*)
Graphite monochromator	*R*_int_ = 0.034
ω & φ scans	θ_max_ = 28.3°, θ_min_ = 2.0°
Absorption correction: multi-scan (*SADABS*; Bruker, 2008)	*h* = −14→14
*T*_min_ = 0.977, *T*_max_ = 0.985	*k* = −16→16
37978 measured reflections	*l* = −21→19

### Refinement

**Table:**

Refinement on *F*^2^	Primary atom site location: structure-invariant direct methods
Least-squares matrix: full	Secondary atom site location: difference Fourier map
*R*[*F*^2^ > 2σ(*F*^2^)] = 0.053	Hydrogen site location: inferred from neighbouring sites
*wR*(*F*^2^) = 0.175	H atoms treated by a mixture of independent and constrained refinement
*S* = 1.04	*w* = 1/[σ^2^(*F*_o_^2^) + (0.070*P*)^2^ + 0.8065*P*] where *P* = (*F*_o_^2^ + 2*F*_c_^2^)/3
5367 reflections	(Δ/σ)_max_ < 0.001
275 parameters	Δρ_max_ = 0.25 e Å^−^^3^
0 restraints	Δρ_min_ = −0.23 e Å^−^^3^

### Special details

**Table:**

Geometry. All e.s.d.'s (except the e.s.d. in the dihedral angle between two l.s. planes) are estimated using the full covariance matrix. The cell e.s.d.'s are taken into account individually in the estimation of e.s.d.'s in distances, angles and torsion angles; correlations between e.s.d.'s in cell parameters are only used when they are defined by crystal symmetry. An approximate (isotropic) treatment of cell e.s.d.'s is used for estimating e.s.d.'s involving l.s. planes.
Refinement. Refinement of *F*^2^ against ALL reflections. The weighted *R*-factor *wR* and goodness of fit *S* are based on *F*^2^, conventional *R*-factors *R* are based on *F*, with *F* set to zero for negative *F*^2^. The threshold expression of *F*^2^ > σ(*F*^2^) is used only for calculating *R*-factors(gt) *etc*. and is not relevant to the choice of reflections for refinement. *R*-factors based on *F*^2^ are statistically about twice as large as those based on *F*, and *R*- factors based on ALL data will be even larger.

### Fractional atomic coordinates and isotropic or equivalent isotropic displacement parameters (Å^2^)

**Table:**

	*x*	*y*	*z*	*U*_iso_*/*U*_eq_	
H1A	0.018 (2)	1.0450 (18)	0.1578 (13)	0.061 (7)*	
O1	0.02902 (16)	0.61978 (10)	0.21324 (9)	0.0619 (4)	
O2	−0.06469 (18)	0.49464 (12)	0.27901 (11)	0.0731 (5)	
N1	0.04764 (16)	0.99163 (12)	0.18907 (10)	0.0436 (4)	
C11	−0.04007 (18)	0.97408 (14)	0.24813 (11)	0.0440 (4)	
H11	−0.1257	0.9608	0.2172	0.053*	
N2	−0.01386 (18)	0.69687 (13)	0.26740 (11)	0.0564 (5)	
C20	0.05405 (18)	0.44768 (15)	0.17305 (12)	0.0456 (4)	
C10	0.0019 (2)	0.87831 (15)	0.30293 (12)	0.0515 (5)	
H10	0.0828	0.8974	0.3378	0.062*	
C7	0.05728 (18)	0.90311 (15)	0.13409 (11)	0.0446 (4)	
H7	−0.0282	0.8857	0.1047	0.054*	
C6	0.14265 (18)	0.92717 (15)	0.07160 (11)	0.0459 (5)	
C9	0.0297 (2)	0.78632 (15)	0.25099 (12)	0.0480 (5)	
C12	−0.04334 (19)	1.07383 (15)	0.29832 (11)	0.0445 (4)	
C19	−0.00232 (19)	0.51999 (15)	0.22822 (12)	0.0478 (5)	
C25	0.10773 (19)	0.48317 (16)	0.10675 (12)	0.0500 (5)	
H25	0.1056	0.5547	0.0946	0.060*	
C24	0.1646 (2)	0.41475 (18)	0.05806 (13)	0.0563 (5)	
C13	−0.1543 (2)	1.13041 (16)	0.29641 (12)	0.0511 (5)	
H13	−0.2295	1.1070	0.2643	0.061*	
C8	0.1099 (2)	0.80955 (16)	0.18605 (14)	0.0562 (5)	
H8A	0.1115	0.7485	0.1508	0.067*	
H8B	0.1968	0.8243	0.2123	0.067*	
C14	−0.1543 (3)	1.22239 (18)	0.34226 (15)	0.0652 (6)	
H14	−0.2294	1.2607	0.3400	0.078*	
C15	−0.0451 (3)	1.25700 (18)	0.39050 (15)	0.0694 (7)	
H15	−0.0463	1.3177	0.4219	0.083*	
C17	0.0669 (2)	1.11168 (17)	0.34612 (14)	0.0606 (6)	
H17	0.1433	1.0756	0.3471	0.073*	
C16	0.0654 (3)	1.20238 (19)	0.39244 (15)	0.0703 (7)	
H16	0.1400	1.2261	0.4250	0.084*	
C1	0.2413 (2)	0.99896 (18)	0.08749 (15)	0.0611 (6)	
H1	0.2542	1.0365	0.1368	0.073*	
C18	−0.0920 (3)	0.85368 (19)	0.36119 (16)	0.0790 (8)	
H18A	−0.1044	0.9153	0.3928	0.119*	
H18B	−0.0587	0.7979	0.3979	0.119*	
H18C	−0.1721	0.8324	0.3295	0.119*	
C22	0.1156 (3)	0.27329 (18)	0.14423 (17)	0.0735 (7)	
H22	0.1193	0.2019	0.1569	0.088*	
C21	0.0567 (2)	0.34152 (16)	0.19135 (15)	0.0590 (6)	
H21	0.0192	0.3164	0.2349	0.071*	
C23	0.1689 (2)	0.30943 (19)	0.07893 (16)	0.0698 (7)	
H23	0.2086	0.2622	0.0481	0.084*	
C5	0.1289 (2)	0.87079 (19)	−0.00125 (13)	0.0608 (6)	
H5	0.0640	0.8213	−0.0126	0.073*	
C4	0.2102 (3)	0.8869 (2)	−0.05753 (14)	0.0768 (8)	
H4	0.1998	0.8479	−0.1062	0.092*	
C2	0.3212 (3)	1.0150 (2)	0.0298 (2)	0.0838 (8)	
H2	0.3864	1.0644	0.0405	0.101*	
C3	0.3058 (3)	0.9595 (3)	−0.04244 (18)	0.0880 (9)	
H3	0.3597	0.9711	−0.0808	0.106*	
C26	0.2245 (3)	0.4555 (3)	−0.01269 (15)	0.0841 (8)	
H26A	0.2596	0.3978	−0.0393	0.126*	
H26B	0.2912	0.5043	0.0079	0.126*	
H26C	0.1609	0.4904	−0.0518	0.126*	

### Atomic displacement parameters (Å^2^)

**Table:**

	*U*^11^	*U*^22^	*U*^33^	*U*^12^	*U*^13^	*U*^23^
O1	0.0913 (11)	0.0369 (7)	0.0691 (9)	−0.0054 (7)	0.0472 (8)	−0.0056 (7)
O2	0.0998 (13)	0.0492 (9)	0.0847 (11)	0.0049 (8)	0.0569 (10)	0.0115 (8)
N1	0.0528 (10)	0.0380 (8)	0.0433 (9)	0.0039 (7)	0.0178 (7)	0.0007 (7)
C11	0.0469 (11)	0.0431 (10)	0.0446 (10)	−0.0001 (8)	0.0154 (8)	−0.0039 (8)
N2	0.0797 (12)	0.0400 (9)	0.0575 (10)	0.0013 (8)	0.0349 (9)	−0.0037 (8)
C20	0.0474 (11)	0.0413 (10)	0.0487 (11)	−0.0028 (8)	0.0102 (9)	−0.0016 (8)
C10	0.0714 (14)	0.0422 (10)	0.0452 (11)	0.0007 (9)	0.0221 (10)	0.0006 (8)
C7	0.0484 (11)	0.0440 (10)	0.0445 (10)	−0.0022 (8)	0.0166 (8)	−0.0035 (8)
C6	0.0493 (11)	0.0487 (11)	0.0420 (10)	0.0070 (9)	0.0145 (8)	0.0073 (8)
C9	0.0611 (12)	0.0387 (10)	0.0486 (11)	0.0028 (9)	0.0217 (9)	0.0021 (8)
C12	0.0528 (11)	0.0408 (10)	0.0429 (10)	0.0008 (8)	0.0169 (9)	−0.0015 (8)
C19	0.0561 (12)	0.0395 (10)	0.0508 (11)	0.0021 (9)	0.0177 (9)	0.0052 (8)
C25	0.0560 (12)	0.0471 (11)	0.0486 (11)	−0.0020 (9)	0.0136 (9)	−0.0011 (9)
C24	0.0520 (12)	0.0665 (14)	0.0519 (12)	−0.0056 (10)	0.0131 (9)	−0.0136 (10)
C13	0.0547 (12)	0.0510 (12)	0.0519 (11)	0.0030 (9)	0.0214 (9)	0.0016 (9)
C8	0.0706 (14)	0.0422 (11)	0.0638 (13)	0.0050 (10)	0.0350 (11)	0.0044 (9)
C14	0.0804 (17)	0.0522 (13)	0.0720 (15)	0.0162 (12)	0.0390 (13)	0.0023 (11)
C15	0.106 (2)	0.0467 (12)	0.0627 (14)	−0.0041 (13)	0.0359 (14)	−0.0135 (11)
C17	0.0611 (14)	0.0520 (12)	0.0677 (14)	0.0033 (10)	0.0084 (11)	−0.0089 (10)
C16	0.0871 (18)	0.0604 (14)	0.0627 (14)	−0.0126 (13)	0.0103 (13)	−0.0135 (11)
C1	0.0604 (14)	0.0604 (13)	0.0679 (14)	−0.0016 (11)	0.0267 (11)	0.0030 (11)
C18	0.127 (2)	0.0534 (14)	0.0716 (16)	0.0007 (14)	0.0616 (16)	0.0000 (11)
C22	0.0898 (18)	0.0391 (12)	0.0947 (19)	−0.0051 (12)	0.0247 (15)	−0.0114 (12)
C21	0.0699 (14)	0.0406 (11)	0.0693 (14)	−0.0073 (10)	0.0192 (11)	−0.0018 (10)
C23	0.0723 (15)	0.0579 (14)	0.0811 (17)	−0.0024 (12)	0.0180 (13)	−0.0283 (12)
C5	0.0667 (14)	0.0741 (15)	0.0440 (11)	0.0095 (11)	0.0158 (10)	−0.0007 (10)
C4	0.0833 (18)	0.108 (2)	0.0433 (12)	0.0332 (17)	0.0227 (12)	0.0103 (13)
C2	0.0679 (16)	0.0896 (19)	0.103 (2)	−0.0024 (14)	0.0420 (15)	0.0255 (17)
C3	0.085 (2)	0.117 (2)	0.0742 (18)	0.0317 (18)	0.0482 (15)	0.0371 (17)
C26	0.0888 (19)	0.110 (2)	0.0610 (15)	0.0015 (16)	0.0345 (14)	−0.0119 (14)

### Geometric parameters (Å, º)

**Table:**

O1—C19	1.345 (2)	C8—H8A	0.9700
O1—N2	1.446 (2)	C8—H8B	0.9700
O2—C19	1.192 (2)	C14—C15	1.364 (4)
N1—C7	1.456 (2)	C14—H14	0.9300
N1—C11	1.468 (2)	C15—C16	1.360 (4)
N1—H1A	0.88 (2)	C15—H15	0.9300
C11—C12	1.516 (2)	C17—C16	1.383 (3)
C11—C10	1.535 (3)	C17—H17	0.9300
C11—H11	0.9800	C16—H16	0.9300
N2—C9	1.273 (2)	C1—C2	1.387 (3)
C20—C21	1.382 (3)	C1—H1	0.9300
C20—C25	1.384 (3)	C18—H18A	0.9600
C20—C19	1.484 (3)	C18—H18B	0.9600
C10—C9	1.505 (3)	C18—H18C	0.9600
C10—C18	1.524 (3)	C22—C23	1.371 (3)
C10—H10	0.9800	C22—C21	1.379 (3)
C7—C6	1.508 (2)	C22—H22	0.9300
C7—C8	1.516 (3)	C21—H21	0.9300
C7—H7	0.9800	C23—H23	0.9300
C6—C5	1.380 (3)	C5—C4	1.380 (3)
C6—C1	1.382 (3)	C5—H5	0.9300
C9—C8	1.500 (3)	C4—C3	1.365 (4)
C12—C13	1.377 (3)	C4—H4	0.9300
C12—C17	1.384 (3)	C2—C3	1.366 (4)
C25—C24	1.386 (3)	C2—H2	0.9300
C25—H25	0.9300	C3—H3	0.9300
C24—C23	1.381 (3)	C26—H26A	0.9600
C24—C26	1.505 (3)	C26—H26B	0.9600
C13—C14	1.390 (3)	C26—H26C	0.9600
C13—H13	0.9300		
			
C19—O1—N2	114.49 (14)	C7—C8—H8B	109.5
C7—N1—C11	114.11 (15)	H8A—C8—H8B	108.1
C7—N1—H1A	106.9 (14)	C15—C14—C13	120.6 (2)
C11—N1—H1A	107.4 (14)	C15—C14—H14	119.7
N1—C11—C12	107.78 (15)	C13—C14—H14	119.7
N1—C11—C10	110.62 (15)	C16—C15—C14	119.8 (2)
C12—C11—C10	112.10 (15)	C16—C15—H15	120.1
N1—C11—H11	108.8	C14—C15—H15	120.1
C12—C11—H11	108.8	C16—C17—C12	121.0 (2)
C10—C11—H11	108.8	C16—C17—H17	119.5
C9—N2—O1	108.23 (15)	C12—C17—H17	119.5
C21—C20—C25	119.53 (19)	C15—C16—C17	120.1 (2)
C21—C20—C19	117.92 (18)	C15—C16—H16	119.9
C25—C20—C19	122.50 (17)	C17—C16—H16	119.9
C9—C10—C18	113.92 (17)	C6—C1—C2	120.0 (2)
C9—C10—C11	110.50 (15)	C6—C1—H1	120.0
C18—C10—C11	111.89 (18)	C2—C1—H1	120.0
C9—C10—H10	106.7	C10—C18—H18A	109.5
C18—C10—H10	106.7	C10—C18—H18B	109.5
C11—C10—H10	106.7	H18A—C18—H18B	109.5
N1—C7—C6	112.08 (16)	C10—C18—H18C	109.5
N1—C7—C8	108.40 (16)	H18A—C18—H18C	109.5
C6—C7—C8	109.50 (15)	H18B—C18—H18C	109.5
N1—C7—H7	108.9	C23—C22—C21	120.8 (2)
C6—C7—H7	108.9	C23—C22—H22	119.6
C8—C7—H7	108.9	C21—C22—H22	119.6
C5—C6—C1	118.31 (19)	C22—C21—C20	119.2 (2)
C5—C6—C7	119.59 (19)	C22—C21—H21	120.4
C1—C6—C7	121.92 (18)	C20—C21—H21	120.4
N2—C9—C8	126.54 (17)	C22—C23—C24	121.2 (2)
N2—C9—C10	117.54 (17)	C22—C23—H23	119.4
C8—C9—C10	115.90 (16)	C24—C23—H23	119.4
C13—C12—C17	118.20 (19)	C4—C5—C6	120.9 (2)
C13—C12—C11	121.42 (18)	C4—C5—H5	119.5
C17—C12—C11	120.36 (18)	C6—C5—H5	119.5
O2—C19—O1	124.49 (18)	C3—C4—C5	120.6 (3)
O2—C19—C20	125.87 (18)	C3—C4—H4	119.7
O1—C19—C20	109.63 (15)	C5—C4—H4	119.7
C20—C25—C24	121.6 (2)	C3—C2—C1	121.1 (3)
C20—C25—H25	119.2	C3—C2—H2	119.4
C24—C25—H25	119.2	C1—C2—H2	119.4
C23—C24—C25	117.7 (2)	C4—C3—C2	119.0 (2)
C23—C24—C26	121.6 (2)	C4—C3—H3	120.5
C25—C24—C26	120.6 (2)	C2—C3—H3	120.5
C12—C13—C14	120.3 (2)	C24—C26—H26A	109.5
C12—C13—H13	119.9	C24—C26—H26B	109.5
C14—C13—H13	119.9	H26A—C26—H26B	109.5
C9—C8—C7	110.72 (16)	C24—C26—H26C	109.5
C9—C8—H8A	109.5	H26A—C26—H26C	109.5
C7—C8—H8A	109.5	H26B—C26—H26C	109.5
C9—C8—H8B	109.5		
			
C7—N1—C11—C12	−177.94 (16)	C19—C20—C25—C24	177.28 (19)
C7—N1—C11—C10	59.2 (2)	C20—C25—C24—C23	−1.5 (3)
C19—O1—N2—C9	175.23 (19)	C20—C25—C24—C26	−178.8 (2)
N1—C11—C10—C9	−48.2 (2)	C17—C12—C13—C14	−0.6 (3)
C12—C11—C10—C9	−168.54 (16)	C11—C12—C13—C14	−179.19 (17)
N1—C11—C10—C18	−176.30 (18)	N2—C9—C8—C7	131.3 (2)
C12—C11—C10—C18	63.4 (2)	C10—C9—C8—C7	−50.5 (3)
C11—N1—C7—C6	176.81 (16)	N1—C7—C8—C9	55.3 (2)
C11—N1—C7—C8	−62.2 (2)	C6—C7—C8—C9	177.81 (17)
N1—C7—C6—C5	−157.34 (18)	C12—C13—C14—C15	−0.9 (3)
C8—C7—C6—C5	82.3 (2)	C13—C14—C15—C16	1.4 (3)
N1—C7—C6—C1	27.6 (3)	C13—C12—C17—C16	1.6 (3)
C8—C7—C6—C1	−92.7 (2)	C11—C12—C17—C16	−179.80 (19)
O1—N2—C9—C8	0.9 (3)	C14—C15—C16—C17	−0.4 (4)
O1—N2—C9—C10	−177.23 (17)	C12—C17—C16—C15	−1.1 (4)
C18—C10—C9—N2	−8.3 (3)	C5—C6—C1—C2	1.9 (3)
C11—C10—C9—N2	−135.2 (2)	C7—C6—C1—C2	177.0 (2)
C18—C10—C9—C8	173.4 (2)	C23—C22—C21—C20	−1.3 (4)
C11—C10—C9—C8	46.4 (3)	C25—C20—C21—C22	1.6 (3)
N1—C11—C12—C13	117.95 (19)	C19—C20—C21—C22	−176.0 (2)
C10—C11—C12—C13	−120.1 (2)	C21—C22—C23—C24	−0.4 (4)
N1—C11—C12—C17	−60.6 (2)	C25—C24—C23—C22	1.7 (4)
C10—C11—C12—C17	61.4 (2)	C26—C24—C23—C22	179.1 (2)
N2—O1—C19—O2	3.1 (3)	C1—C6—C5—C4	−1.1 (3)
N2—O1—C19—C20	−176.11 (16)	C7—C6—C5—C4	−176.3 (2)
C21—C20—C19—O2	−13.2 (3)	C6—C5—C4—C3	−0.3 (4)
C25—C20—C19—O2	169.3 (2)	C6—C1—C2—C3	−1.2 (4)
C21—C20—C19—O1	165.91 (19)	C5—C4—C3—C2	1.0 (4)
C25—C20—C19—O1	−11.5 (3)	C1—C2—C3—C4	−0.3 (4)
C21—C20—C25—C24	−0.1 (3)		

### Hydrogen-bond geometry (Å, º)

**Table:**

*D*—H···*A*	*D*—H	H···*A*	*D*···*A*	*D*—H···*A*
C3—H3···O2^i^	0.93	2.59	3.485 (3)	160

Symmetry code: (i) *x*+1/2, −*y*+3/2, *z*−1/2.
